# Visual language transformer framework for multimodal dance performance evaluation and progression monitoring

**DOI:** 10.1038/s41598-025-16345-2

**Published:** 2025-08-20

**Authors:** Lei Chen

**Affiliations:** Art College, Chengdu Sport University, Chengdu, 610041 China

**Keywords:** Deep learning, Transformer, Graph convolutional network, Dance performance monitoring, Multimodal analysis, Computational science, Computer science, Information technology, Software

## Abstract

Dance is often perceived as complex due to the need for coordinating multiple body movements and precisely aligning them with musical rhythm and content. Research in automatic dance performance assessment has the potential to enhance individuals’ sensorimotor skills and motion analysis. Recent studies on dance performance assessment primarily focus on evaluating simple dance movements using a single task, typically estimating final performance scores. We propose a novel transformer-based visual-language framework for multi-modal dance performance evaluation and progression monitoring. Our approach addresses two core challenges: the learning of feature representations for complex dance movements synchronized with music across diverse styles, genres, and expertise levels, and capturing the multi-task nature of dance performance evaluation. To achieve this, we integrate contrastive self-supervised learning, spatiotemporal graph convolutional networks (STGCN), long short-term memory networks (LSTM), and transformer-based text prompting. Our model evaluates three key tasks: (i) multilabel dance classification, (ii) dance quality estimation, and (iii) dance-music synchronization, leveraging primitive-based segmentation and multi-modal inputs. During the pre-training phase, we utilize contrastive loss to capture primitive-based features from complex dance motion and music data. For downstream tasks, we propose a transformer-based text prompting approach to conduct multi-task evaluations for the three assessment objectives. Our model outperforms in diverse downstream tasks. For multilabel dance classification, our model achieves a score of 75.20, representing a 10.25% improvement over CotrastiveDance, on the dance quality estimation task, the proposed model achieved a 92.09% lower loss on CotrastiveDance. For dance-music synchronization, our model excels with a score of 2.52, outperforming CotrastiveDance by 48.67%.

## Introduction

Dance is a universal art form that serves as a medium of artistic expression and holds cultural, social, and therapeutic significance. Its complexity lies in the precise coordination of physical movements, timing, and expression, often tailored to diverse styles, genres, and rhythms. Despite its widespread importance, systematic methods for evaluating dance performance remain underdeveloped, creating a gap in assessing and improving performance quality objectively^1^. The evaluation of dance proficiency involves intricate processes, including the analysis of movement dynamics, rhythm synchronization, and aesthetic expression, all of which require substantial expertise and experience. This creates a pressing need for automated frameworks that can mimic expert evaluation while offering scalability and accessibility.

**Fig. 1:**
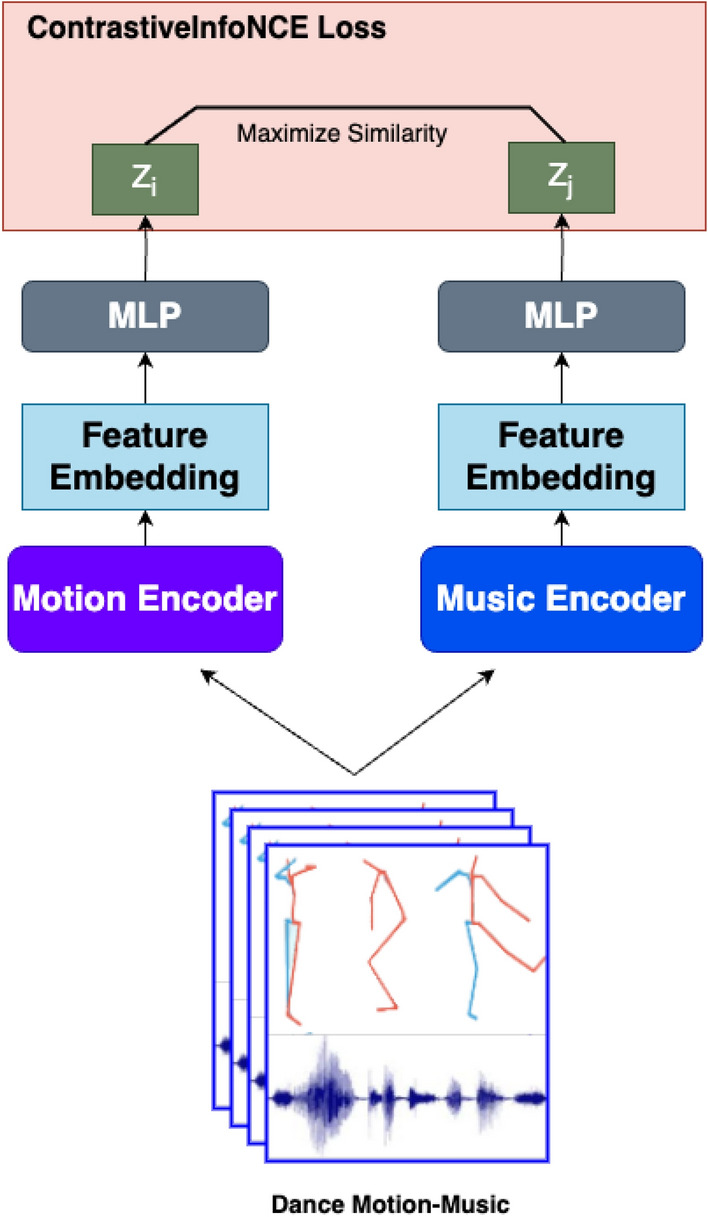
The diagram provides a high-level view of the contrastive self-supervised framework for dance motion-music feature representation. It employs two encoders: a motion encoder and a music encoder, processing respective features from the input data. These features are embedded into a shared space, with MLP modules projecting the embeddings, $Z_i$ (motion) and $Z_j$ (music), into the same latent space for comparison. This framework learns robust motion-music alignment for downstream tasks like performance evaluation and rhythm synchronization.

Skilled dancers execute intricate movements and synchronize them with musical beats, a proficiency acquired through extensive practice and training. However, access to professional training is often costly and constrained by time and location. To address this limitation, there is potential for developing a multi-task dance performance assessment model that continuously evaluates a dancer’s performance quality^2^. Most previous research on Action Quality Assessment (AQA) and Skill Assessment (SA) has predominantly focused on areas such as surgical tasks^3^, Olympic sports^4^, and everyday human activities^5^, with limited emphasis on dance performance evaluation. Recent studies on dance and music analysis have largely concentrated on tasks like dance motion generation^6^ and motion-music synthesis^1^, rather than the integrated assessment of dance performance. Other methods of dance motion analysis^7^ have focused on simplified, controlled conditions, often neglecting music features or offering only final performance scores. Therefore, two key challenges emerge in the automatic assessment of dance performance: (i) learning feature representations for complex dance movements and music, where representations vary significantly across different styles, genres, choreographies, and expertise levels; and (ii) capturing the multi-task nature of dance performance evaluation, which involves interpreting diverse multimedia content, such as identifying elements in the dance sequence, scoring each element, and analyzing the relationship between motion and music.

Dance performance, however, presents unique challenges due to its inherently multimodal nature, requiring a deep understanding of both motion and music features. Furthermore, the variability across dance styles, choreographies, and expertise levels introduces additional complexity^4^. Addressing these challenges not only fills a critical gap in the field of motion analysis but also has the potential to revolutionize professional training, democratize access to dance education, and contribute to broader applications such as rehabilitation, entertainment, and cultural preservation.

Primitive-based segmentation plays a pivotal role in our proposed framework by breaking down dance sequences into smaller, rhythmically-aligned units known as primitives. This segmentation enables the model to capture the intricate relationship between dance movements and the rhythmic structure of the music at a granular level. By focusing on primitives, the framework can evaluate subtle details of motion synchronization, transitions, and rhythmic accuracy that are often overlooked in holistic performance analysis. The Eight-Beats Segmentation (EBS) method is employed to systematically divide dance sequences into rhythmic intervals aligned with musical beats. This alignment is critical for extracting temporal and spatial features that encapsulate the synergy between motion and music, providing a robust foundation for downstream tasks such as multilabel classification, quality estimation, and synchronization analysis.

**Fig. 2:**
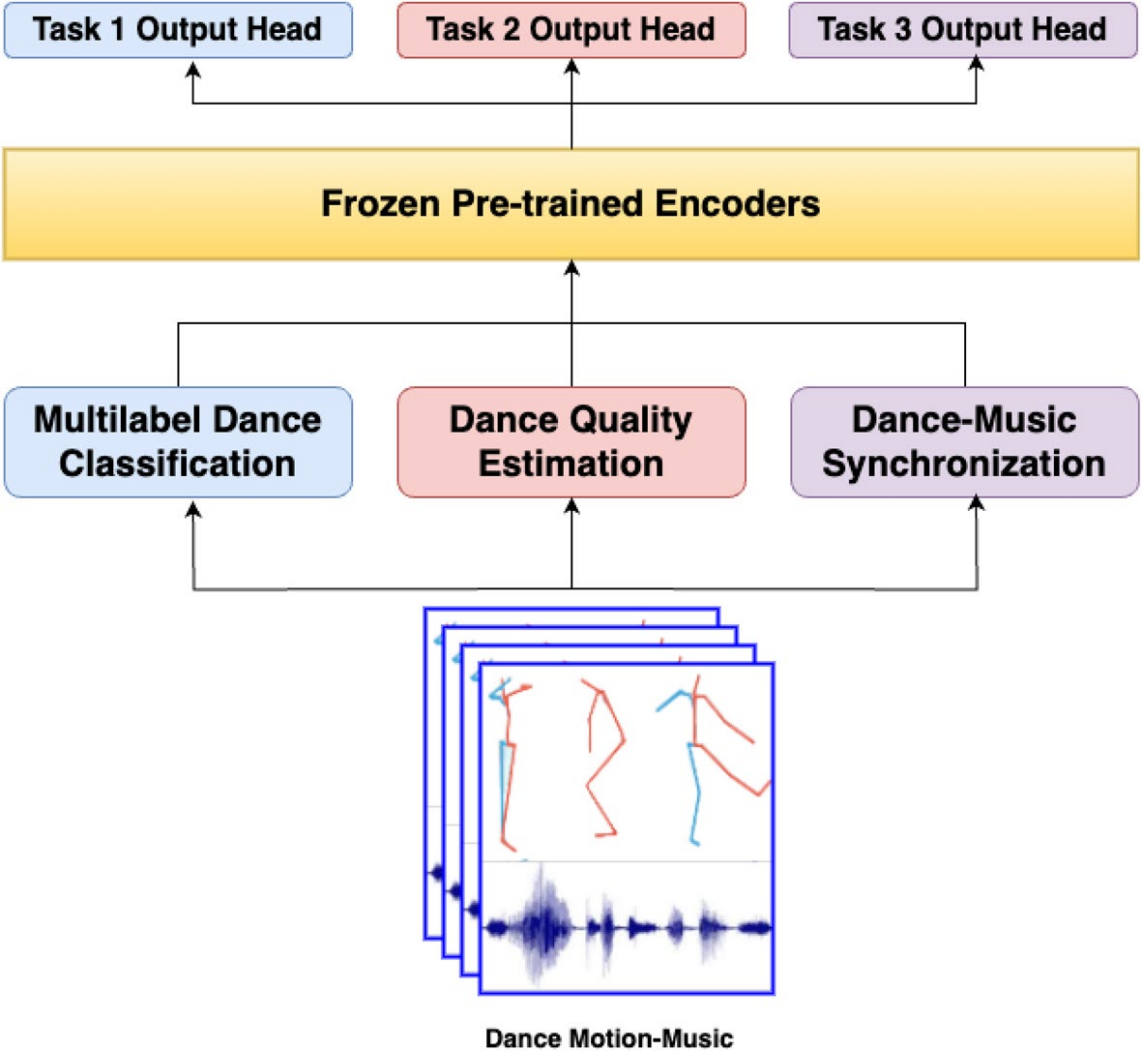
The diagram illustrates the architecture for multi-task dance performance evaluation, consisting of three tasks: (i) Multilabel Dance Classification, (ii) Dance Quality Estimation, and (iii) Dance-Music Synchronization. Frozen pre-trained encoders extract features from dance motion-music input, which are then fed into three task heads. Task 1 classifies dance choreography, genres, and expertise levels. Task 2 estimates dance quality through performance scoring. Task 3 aligns motion sequences with musical beats for synchronization evaluation. The architecture effectively unifies dance and music feature analysis, leveraging pre-trained encoders for streamlined performance assessment.

Our proposed method tackles the aforementioned challenges by incorporating advanced machine learning techniques tailored for complex, real-world professional dance training environments. The framework leverages the diversity of dance genres, choreographies, and expertise levels, ensuring its robustness in handling the intricate nuances of dance performances. Figures 1 and  2 illustrate the self-supervised learning pipeline of our approach, which focuses on extracting meaningful representations from both motion and music data. At the core of our method is a contrastive learning framework that effectively learns feature representations of primitive-based dance sequences. Primitive-based segmentation breaks down the dance sequences into smaller, rhythmically-aligned units, allowing the model to capture fine-grained details of movement and their synchronization with musical elements. This segmentation is crucial for assessing a dancer’s performance not just holistically but also at the micro-level, ensuring that subtle transitions and rhythmic accuracy are evaluated. The contrastive learning mechanism optimizes the model to distinguish between positive (synchronized) and negative (unsynchronized) pairs of motion and music features, thus enabling the framework to generalize across dancers with varying levels of expertise. By training on such pairs, the model learns to recognize the complex temporal and spatial relationships that define dance proficiency, while accounting for variations in skill levels. This allows for a more granular and nuanced evaluation of dance performance, addressing both the aesthetic quality of movements and their rhythmic synchronization with music. Our approach emphasizes domain adaptability through contrastive learning, ensuring that the model is capable of performing well in dynamic environments and across different dance styles and genres without the need for extensive labeled data. These dance sequences encompass a wide range of music genres and choreographies. Rather than evaluating the entire dance sequence, as in previous work^7,8^, our model is trained using motion and music primitives. Each motion and music sequence is segmented into primitives using the Eight-Beats segmentation (EBS) method, which captures the rhythmic structure in the data^9^. We utilize SpatioTemporal Graph Convolutional Networks (STGCN)^10^ and Long Short-Term Memory network (LSTM)^11^ as encoders to learn the features of these dance primitives.

Once the motion and music primitive features are extracted, we apply a transformer-based text prompting technique^12^ to assess the dance performance across three downstream tasks. The challenge of evaluating diverse dance genres and choreographies emerging globally requires adaptability. Continuously training new models for incoming data is impractical, and providing accurate predictions often demands large datasets of dance sequences under similar conditions (e.g., the same choreography, music, or time steps), which can be difficult to gather and label. To address the inherent complexities associated with evaluating diverse and unseen dance motions and music, we incorporate prompt tuning into our pre-trained model, a technique that has proven to be more efficient and effective than conventional fine-tuning methods in handling downstream tasks. Prompt tuning leverages pre-trained knowledge while requiring minimal adjustments to the model parameters, allowing it to adapt to new tasks by simply modifying the input prompt. This approach significantly reduces the need for extensive retraining, particularly in scenarios where acquiring large amounts of labeled data is impractical, such as professional dance evaluation across diverse genres and styles. Our experimental results provide compelling evidence that prompt tuning offers superior performance compared to traditional fine-tuning methods when applied to three critical downstream tasks in dance performance assessment: (i) multilabel dance classification, (ii) dance quality estimation, and (iii) dance-music synchronization. By leveraging prompt tuning, our model can generalize more effectively to diverse and previously unseen dance sequences and musical inputs, demonstrating enhanced adaptability and performance stability.

- We present a contrastive self-supervised learning framework designed to capture and represent the features of primitive-based motion and music sequences.
- We introduce a transformer-based text prompting mechanism, which is used to artistically model the downstream multi-task dance performance assessment process.
- Proposed multi-modal framework is evaluated on three downstream tasks including (i) multilabel dance classification, involving the identification of dance motion primitives; (ii) dance quality estimation, focusing on estimating quality score distributions; and (iii) dance-music synchronization, which involves regressing the alignment rate between music and motion.

The literature review is presented in “Literature review”. The proposed multi-modal framework for dance performance assessment and problem formulation is explained in “Method”. The dataset, experimental setup, and performance comparisons are presented in “Experiment and result”, while “Conclusion” summarises the study and concludes it with a future direction.

## Literature review

### Human motion analysis

The study of human motion has seen significant advancements in areas such as motion generation^13^, rehabilitation^14^, and sports performance analysis^15^. A particular area of focus dance motion analysis, which aims to automatically and quantitatively assess the quality of dance performances has gained increasing attention, primarily due to the advancements in motion analysis^16^ and multimedia signal processing technologies^17^. However, most existing methodologies are limited to evaluating simple dance motions based on specific choreographies and expertise levels, which limits their generalizability for real-world applications in assistive technologies. Recent work^7^ introduced a self-supervised approach to evaluate dancers’ expertise levels across different dance genres and choreographies. Nonetheless, other crucial tasks such as dance-music synchronization and dance quality estimation require further exploration.

### Quality assessment

In addition to motion analysis, assessing the skill level associated with human motion is integral to evaluating dance performance. While a large portion of existing AQA research has focused on surgical proficiency^18^ or evaluating Olympic-level sports performance^19^, little attention has been paid to dance performance assessment. These approaches have demonstrated effectiveness by concentrating on feature extraction for surgical or sports tasks. However, applying these methods to dance assessment remains challenging, as they often focus solely on motion feature representation and overlook the correlation between motion and other key elements, such as musical rhythm.

### Self-supervised learning

Self-supervised learning (SSL) has shown remarkable success across a wide range of applications, including computer vision, natural language processing, and audio analysis^20,21^. Its key advantage is the ability to leverage large amounts of unlabeled data to learn meaningful feature representations, reducing reliance on costly labeled datasets. This is especially valuable in domains like human motion analysis, where obtaining accurate labeled data is challenging. In human motion analysis, SSL enables robust and generalized feature learning by discovering patterns within the data without explicit labels. Traditional supervised methods often rely on annotated data, introducing biases and limiting generalizability. SSL, on the other hand, uses pretext tasks like contrastive learning to train models that distinguish between similar and dissimilar motion sequences, capturing complex spatial and temporal dependencies in motion data. This approach significantly enhances the model’s ability to generalize across different motion styles, expertise levels, and contexts, making it highly effective for tasks like action recognition, skill assessment, and performance evaluation. By focusing on intrinsic motion features, SSL-based models offer greater scalability and adaptability, pushing the boundaries of motion analysis without the constraints of labeled data. Recent works in this area have utilized contrastive loss to learn similarities between sample pairs. For instance,^22^ demonstrated that contrastive self-supervised learning enables models to achieve state-of-the-art performance in image classification, proving that well-designed data augmentation strategies can result in robust feature representations.

While SSL techniques have been extensively applied in visual tasks, the application of such methods to human 3D-skeleton data remains comparatively underexplored. Prior work, such as that by^23^, has demonstrated the potential of leveraging human 3D-skeleton data, introducing skeleton augmentation techniques to enhance the performance of human action recognition. However, the full potential of contrastive learning for 3D-skeleton-based motion data has yet to be realized in more complex domains like dance performance. In this study, we adopt a contrastive learning framework to learn robust representations of 3D-skeleton dance motion data synchronized with music features. Our approach focuses on mapping the dance motion and music features into a shared latent space, where the temporal and spatial dynamics of the dance motion, along with rhythmic and acoustic elements of the music, are preserved. By doing so, we facilitate the performance of three key downstream tasks: (i) multilabel dance classification, which involves the identification of dance genres, choreographies, and expertise levels; (ii) dance quality estimation, focusing on the prediction of performance scores; and (iii) dance-music synchronization, which measures the alignment between motion and musical rhythm^14^. The integration of contrastive learning enables the model to effectively distinguish between different dance sequences, capturing both intra- and inter-class variations in dance movements and their corresponding musical features. This approach significantly enhances the model’s capacity to generalize to unseen dance performances, demonstrating the efficacy of contrastive learning in multimodal contexts involving 3D-skeleton data and music.

### Prompt tuning

The success of SS has significant advancements in improving the generalization capabilities of pre-trained models, especially for downstream tasks. This research emphasis is particularly critical in scenarios requiring few-shot or zero-shot learning, where models are expected to perform well with minimal labeled data or in the absence of task-specific training data. By leveraging SSL, models can capture robust feature representations that enhance their adaptability to new, unseen tasks. This research direction has been exemplified by models such as Generative Pre-trained Transformer-3 (GPT-3)^24^. Among the various techniques aimed at improving generalization, prompt tuning has emerged as one of the most prominent approaches. Inspired by the recent advancements in prompting techniques in both vision and language models^12,24^, we adapt prompt tuning to our dance-music multimodal model. This adaptation allows the model to dynamically adjust to diverse, previously unseen dance motions and musical inputs with minimal modifications. By incorporating prompt tuning, we leverage the pre-trained model’s extensive prior knowledge while introducing task-specific prompts that guide the model towards new tasks without the need for full re-training. This approach addresses key challenges in generalizing dance performance assessment across multiple styles, genres, and choreography variations. It also significantly reduces the computational complexity typically associated with fine-tuning. Through prompt tuning, the model efficiently adapts to new dance and music combinations.

## Method

The proposed framework integrates multiple state-of-the-art techniques in a cohesive manner to enhance multi-modal dance performance evaluation. At its core, the system leverages a *contrastive self-supervised learning (SSL)* approach to learn robust feature representations from unlabelled motion and music data. The *Spatio-Temporal Graph Convolutional Network (STGCN)* and *Long Short-Term Memory (LSTM)* encoders are used in tandem to model the spatial-temporal dynamics of skeletal motion and the temporal progression of music features. STGCN captures the spatial relationships and temporal dependencies within skeletal motion sequences, while LSTM complements this by learning sequential patterns in both motion and music. These embeddings are further refined and aligned using the SSL framework with the InfoNCE loss, ensuring meaningful feature extraction across modalities. To bridge these embeddings with downstream tasks, the model incorporates a *transformer-based text prompting mechanism*, which integrates textual descriptions (e.g., genres, choreography, or expertise level) as context. This prompting technique enables seamless adaptation of pre-trained representations to specific evaluation tasks by aligning textual, motion, and music embeddings within a unified framework.

**Fig. 3:**
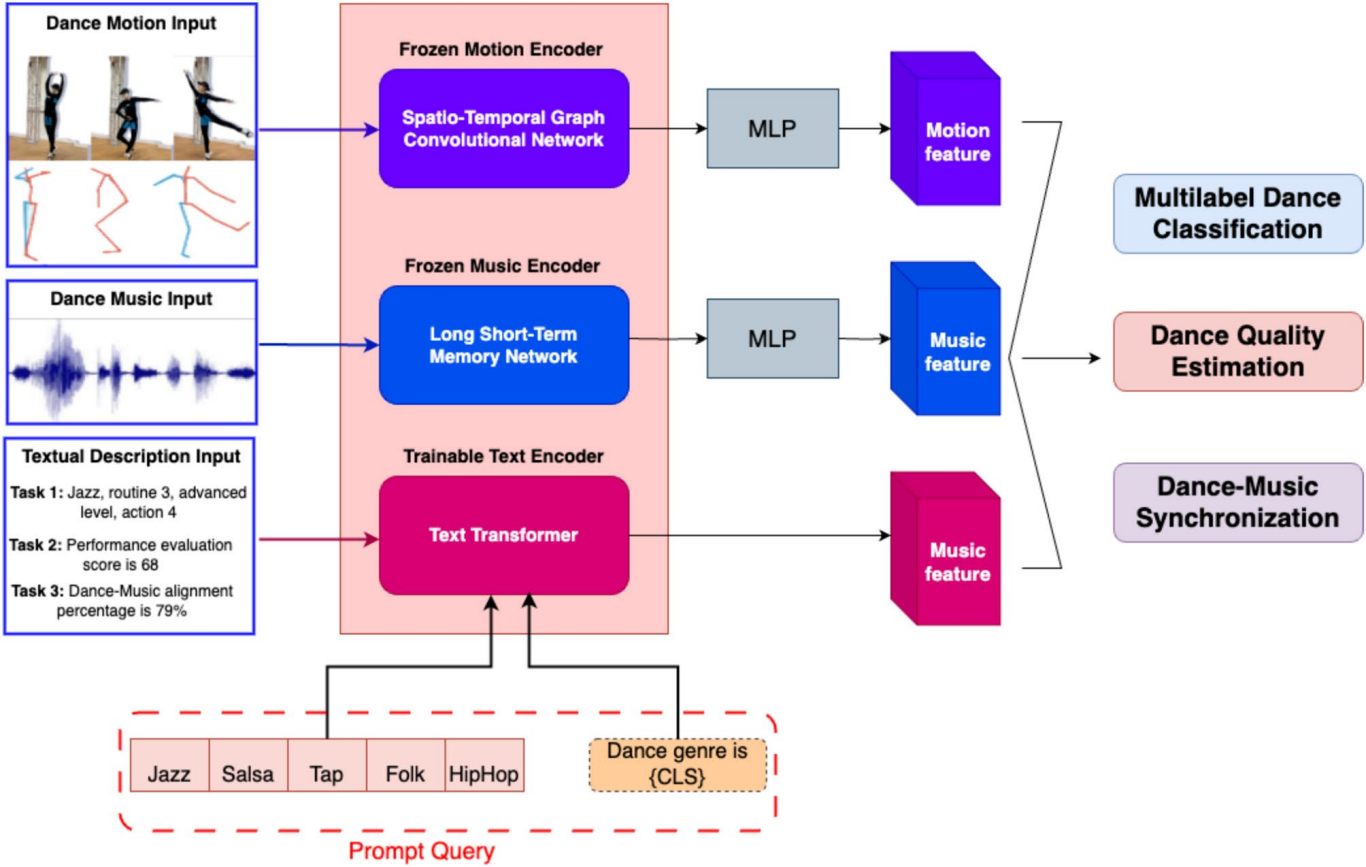
The diagram presents a multi-modal framework for dance performance evaluation using motion, music, and text inputs. It includes three components: (i) a frozen STGCN for motion encoding, (ii) a frozen LSTM network for music encoding, and (iii) a trainable Transformer-based text encoder. These encoders generate features for three tasks: Multilabel Dance Classification, Dance Quality Estimation, and Dance-Music Synchronization. Inputs include dance motion sequences, music waveforms, and text descriptions. MLPs process the features and prompt queries to refine textual inputs for dynamic task evaluation.

The synergy between these components ensures that the model captures intricate interactions between motion, music, and textual information, enhancing its ability to generalize across diverse tasks. Figure 3 presents a detailed overview of the proposed multi-modal framework for dance performance evaluation, which combines motion, music, and textual data to achieve high-level dance analysis. The framework consists of three key inputs: (i) Dance Motion Input, representing skeletal motion data derived from dance sequences, (ii) Dance Music Input, which provides the corresponding audio features, and (iii) Textual Description Input, containing task-related metadata for specific evaluation tasks such as multilabel dance classification, dance quality estimation, and dance-music synchronization.

### Problem formulation

In Fig. 3, we introduce an SSL framework for multi-task, primitive-based dance performance evaluation. The framework is designed to address the complexity of evaluating dance performances by decomposing them into manageable components, or primitives, and conducting assessments across multiple tasks. The primary goal of this framework is to systematically evaluate the quality of input dance sequences through the execution of three critical tasks: (i) Multilabel Dance Classification, (ii) Dance Quality Estimation, and (iii) Dance-Music Synchronization.

Initially, we employ a combination of STGCN and LSTM encoders to capture the spatial-temporal dependencies in both dance motion and music features. This is achieved through a contrastive learning framework, leveraging unlabelled data and optimizing the feature representations with the Information Noise-Contrastive Estimation (InfoNCE) loss function. The model’s input consists of a set of sequences, $S = \{S_{\text {motion}}, S_{\text {music}}\}$, representing both the skeletal motion data of the dancer and the corresponding music features. The STGCN is responsible for extracting the spatial-temporal features from the skeletal data, while the LSTM encoder captures the temporal progression of both the dance motions and the music. Once the dance motion and music embeddings are extracted, we proceed with multi-task downstream knowledge transfer on unseen data.

This is achieved using a transformer-based text prompt tuning methodology, which fine-tunes the pre-trained model to adapt to new downstream tasks. Unlike traditional fine-tuning approaches, this method introduces text prompts that describe the specific attributes of the input data, such as dance genre, choreography, or performance quality, serving as cues for the model to align the learned representations with the task at hand. By leveraging textual prompts as additional context, the model can efficiently transfer knowledge and adapt to diverse evaluation tasks, enhancing its flexibility and performance across different domains. The transformer architecture allows for the seamless integration of these prompts with the learned representations of motion and music, enabling the model to generalize effectively across diverse tasks. This technique improves the model’s capacity to handle complex and unfamiliar dance sequences.

### Pre-training

#### Eight-beats segmentation

Primitive-based segmentation is implemented using the Eight-Beats Segmentation (EBS) method to break down dance sequences into rhythmically aligned units, or primitives. Each primitive corresponds to an interval of eight musical beats, ensuring that the segmented dance motions mirror the rhythmic patterns in the music. This segmentation is essential for capturing fine-grained details of motion-music synchronization, as it allows the model to isolate and analyze dynamic transitions, subtle gestures, and rhythmic accuracy. By structuring the data in this way, EBS facilitates more effective feature extraction for learning complex temporal and spatial dependencies across both modalities. The segmented primitives serve as the foundation for downstream tasks, enabling the model to evaluate performance at a granular level while maintaining a focus on the interplay between motion and music.

Prior to training, we employ an EBS^25^ technique to identify the motion and music primitives within the input dance sequences. The Eight-Beats approach involves breaking down and counting dance movements^8,9^. In this approach, the dance sequences are systematically segmented into discrete motion primitives, aligned with the rhythmic structure of the accompanying music. The segmentation process follows the Eight-Beats method, where each musical sequence is divided into intervals corresponding to every eight beats of the music. This partitioning captures the temporal alignment between the dancer’s movements and the underlying musical rhythm, providing a granular representation of the performance. Specifically, the musical sequence is divided into three distinct groups, each containing eight beats, as denoted by the pink dashed lines. This segmentation method ensures that the rhythmic patterns of the music are closely mirrored by the corresponding motion primitives, facilitating a more detailed analysis of the synchronization between the dancer’s movements and the music. By employing this structured approach, the segmentation preserves both the temporal and rhythmic nuances of the dance, allowing the model to capture complex motion-music correlations for downstream tasks such as performance evaluation and rhythm synchronization.

The beats are detected and segmented using the Librosa audio signal processing library^26^, which facilitates the identification of rhythmic structures within the music. Following this, the corresponding dance motions are similarly divided into three groups of motion primitives, ensuring synchronization with the identified musical beats. From the analysis, we observe that the dance movements in the first group display contrasting patterns compared to those in the third group, highlighting the dynamic nature of the choreography across the sequence. This segmentation approach, termed the Eight-Beats method, proves effective in isolating meaningful and rhythmically aligned dance movements. It captures the intricate relationship between choreography and musical content, making it a robust tool for analyzing motion in conjunction with music. This technique allows the model to learn from the segmentation and analyze dance performances in a granular manner, which is essential for multi-modal tasks such as dance quality evaluation and dance-music synchronization.

#### Motion and music encoders

In this study, we employ a Graph-Based Neural Network^27^, specifically the STGCN^10^, to extract both the spatial and temporal features from human motion. The input to the graph consists of sequences of dance motion $m_i^k$, where *i* represents the index of the body joints and *k* corresponds to the frame number. This encoder is expressed as:

1 $$\begin{aligned} p_i = STGCN(m_i^k) = \phi (m_i^k) \end{aligned}$$

For the extraction of music features, the raw music is first pre-processed to derive acoustic features, as these are expected to provide more relevant information than the raw data. The acoustic features are extracted using the librosa library^26^. Specifically, we focus on five categories of features $\mathcal {F}(a_i) = \{a_0, a_1,..., a_4\}$, which include the Mel-frequency cepstral coefficients (MFCC)^28^, MFCC-delta, constant-Q chromagram, tempogram, and onset strength. Following this extraction, we utilize an LSTM-based encoder $\psi (\cdot )$ to model the acoustic features^11^, formulated as:

2 $$\begin{aligned} q_i = LSTM(a_i) = \psi (a_i) \end{aligned}$$

The motion and music embeddings, denoted as $z_i$, are subsequently obtained by concatenating the output of the two encoders and processed through a multi-layer perceptron (MLP).

#### ContrastiveInfoNCE loss

Once the motion and music embeddings $z_i$ are obtained, the InfoNCE loss^29^ is employed as it effectively facilitates the learning of feature representations and enhances generalizability across various domains^11^. The loss function operates by contrasting the distance between positive and negative sample pairs in the network’s output space, encouraging the model to bring similar (positive) pairs closer together and push dissimilar (negative) pairs further apart in the latent space. During training, augmented pairs are generated from each sample, producing a total of 2*N* data points, where *N* represents the number of samples in the mini-batch. This augmentation helps the model to generalize better by learning robust feature representations across varied instances of the data, facilitating improved performance across downstream tasks. As described by^8^, apart from the positive sample, the remaining $2(N-1)$ samples within the mini-batch are considered negative. When (*i*, *j*) forms a positive pair, the loss is defined as:

3 $$\begin{aligned} \ell _{i,j} = -\log \frac{\exp (\text {sim}(z_i, z_j)/\tau )}{\sum _{r=1}^{2N} \mathbb {1}_{[r\ne i]} \exp (\text {sim}(z_i, z_r)/\tau )} \end{aligned}$$

where the indicator function $\mathbb {1}_{[r \ne i]} \in \{0, 1\}$ equals 1 if $r \ne i$. From the results, it can be inferred that a lower contrastive loss value signifies that positive sample pairs (i.e., corresponding dance and music segments) have been effectively mapped to similar or closer representations within the latent space. Conversely, negative sample pairs (i.e., non-corresponding dance and music segments) are pushed apart, being mapped to more dissimilar or distant representations. This behavior is crucial for ensuring that the model learns to differentiate between synchronized and non-synchronized dance-music pairs. The generation of these positive and negative pairs is achieved through the use of data augmentation techniques, which introduce variations in the training data to enhance the model’s robustness and generalizability. These techniques augment the dance and music sequences, creating multiple representations of the same motion-music relationship while preserving the essential temporal and rhythmic alignment. By optimizing the contrastive loss, the model becomes more proficient at capturing fine-grained dependencies between dance motions and musical rhythms, a critical factor in tasks such as dance-music synchronization and dance quality estimation.

### Transformer-based text prompting

Figure 3 demonstrates the proposed downstream text-prompting workflow. In this approach, the content of the test dance sequences (e.g., dance genres, expertise levels, or performance scores) is initially converted into text descriptions that serve as input to the model. A transformer encoder is employed to derive feature embeddings from this raw textual input, capturing the semantic meaning of the descriptions. Both the text input and the encoded embeddings act as prompts, guiding the model’s predictions. The text features generated by the transformer are then concatenated with the corresponding motion and music features extracted from the pre-trained encoders. In this framework, the integrated motion-music features, along with textual descriptions, are processed to generate task-specific predictions, which are subsequently compared against the ground truth for each downstream task using a task-agnostic loss function. A critical aspect of this approach is that the pre-trained parameters of the motion and music encoders remain fixed throughout the downstream evaluation phase. This ensures that only the text encoder and the final layer of the MLP are fine-tuned, allowing the model to adapt to new tasks while preserving the robustness of the learned motion-music representations. The model effectively fuses textual descriptions with the corresponding motion and music data, enabling enhanced performance across multiple downstream tasks. Figure 3 illustrates examples of input text descriptions utilized for the evaluation tasks, such as genre, choreography, expertise level, and specific dance movements, which are mapped to the encoded features to produce task-specific predictions. This approach enhances the flexibility of the model in handling complex, multimodal inputs while optimizing performance across various assessment tasks. For instance, in the context of the first task, the text encoder might receive a structured input such as *Ballet, choreography 1, beginner, and move 2*. This text serves as a verbal description of the relevant attributes within the sequence. Such descriptions are processed by the model to align with corresponding motion and music features.

### Downstream tasks

**Fig. 4:**
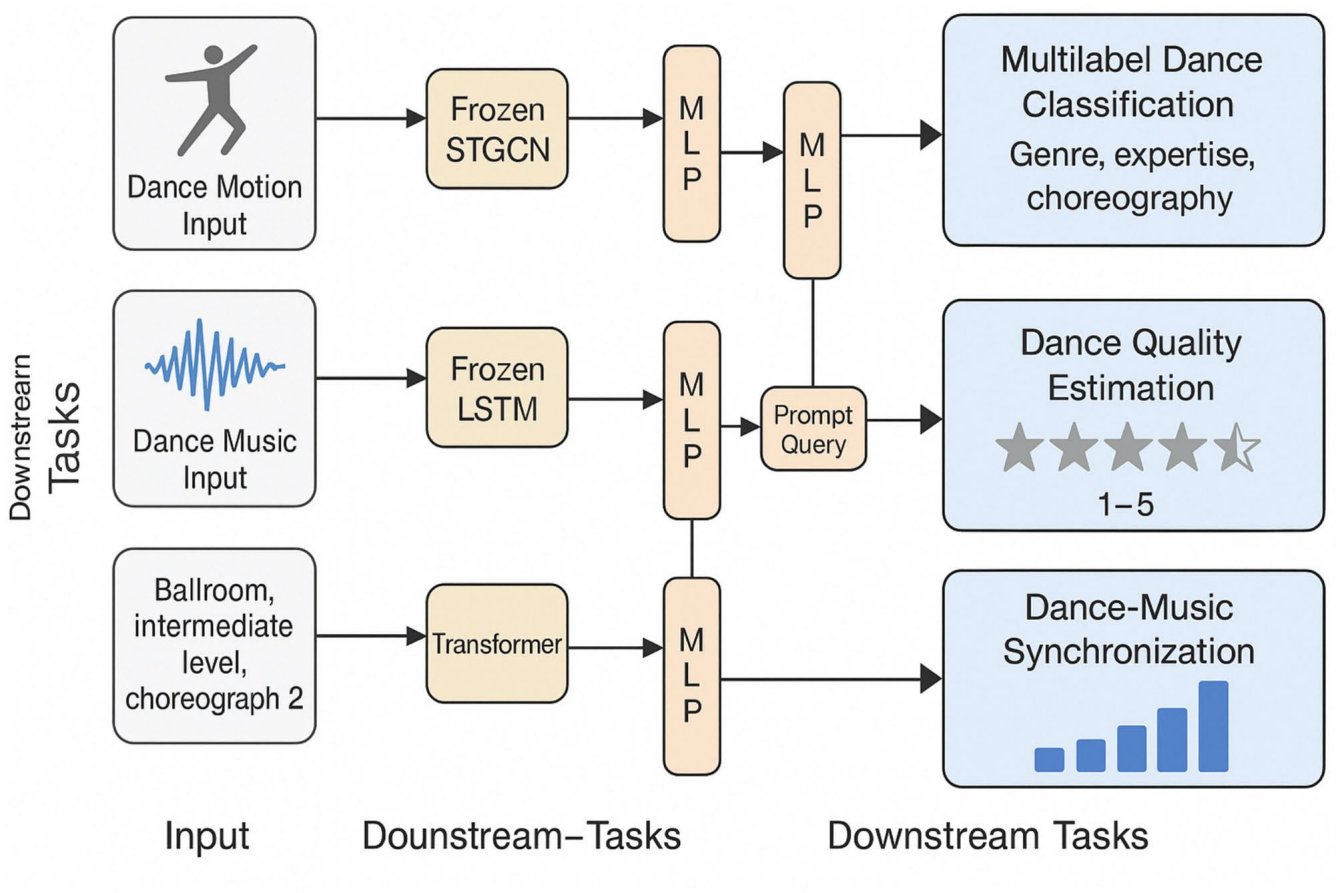
Workflow diagram illustrating the three downstream tasks of the proposed multi-modal dance performance evaluation framework. The diagram shows how extracted motion, music, and textual features are fed into distinct task-specific branches: (i) Multilabel Dance Classification, which identifies genre, choreography, expertise level, and primitive motions using cross-entropy loss; (ii) Dance Quality Estimation, which predicts a probabilistic performance score distribution using Gaussian regression with aleatoric uncertainty modeling; and (iii) Dance-Music Synchronization, which quantifies rhythmic alignment between motion and music through intensity correlation and MSR, optimized via mean squared error.

Evaluating dance performance requires a professional analysis of multimodal data. In this work, we address this by formulating it into three tasks: (i) multilabel dance classification, (ii) dance quality estimation, and (iii) dance-music synchronization. During the downstream phase, unseen test dance sequences are input into the pre-trained model, and each task is evaluated using a text prompting approach as shown in Fig. 4.

#### Multilabel dance classification

The task of multilabel dance classification entails the identification and categorization of multiple attributes associated with dance sequences, including *dance genres*, *choreographies*, *expertise levels*, and *motion primitives*. This task is inherently a *multi-label classification problem*, where each dance sequence may be associated with multiple labels across these dimensions. Specifically, the dataset used for training consists of 20 distinct choreographies, 3 levels of dancer expertise, and 3 primitive motions. This combination results in 180 unique classes, computed as $20 \times 3 \times 3 = 180$, which capture the diverse range of possible configurations within the dataset. To effectively classify the dance vocabulary, we employ a *cross-entropy loss function* during network training. Cross-entropy loss is particularly suited for multi-label classification tasks, as it quantifies the divergence between the predicted label distribution and the true label distribution. By optimizing this loss, the model learns to assign appropriate labels to dance sequences, accurately distinguishing between variations in genres, choreographies, expertise levels, and motion primitives. This approach ensures that the network can generalize well across the broad spectrum of dance elements, facilitating robust performance in identifying complex dance patterns across multiple labels.

#### Dance quality estimation

Task 2 addresses the problem of dance performance evaluation by formulating it as a *score distribution prediction* task. In this approach, each dance sequence within the dataset is annotated with a Quality score, which serves as the ground truth for training and evaluation. These scores are provided by professional dancers, ensuring that the annotations are both expert-driven and reflect real-world dance assessment criteria. However, given the subjective nature of dance performance scoring, individual assessments can introduce variability and bias into the dataset. To account for this inherent uncertainty, our method does not focus on predicting a single, exact score. Instead, we model the problem as a *distributional prediction*, where the model learns to map the extracted dance features to a *probabilistic score distribution*. This allows the system to capture the range of possible scores that may be assigned to a given performance, thus incorporating *aleatoric uncertainty* type of uncertainty that arises from the data itself due to ambiguities or noise. By predicting a distribution rather than a single score, the model can better account for the subjectivity and variability introduced by different evaluators during the labeling process. This approach not only reduces the impact of individual biases but also provides a more nuanced and flexible evaluation framework. It reflects the variability inherent in human judgments and allows the system to express confidence in its predictions by modeling the variance within the score distribution. Such a probabilistic treatment of dance performance evaluation is critical for tasks that involve complex, subjective assessments, as it improves robustness and provides more informative output. This method enables a more accurate representation of the true range of scores a performance might receive, leading to more reliable and interpretable results in downstream tasks. A Gaussian distribution is used to model the predicted score and its corresponding variance. Thus, the loss function is formulated as follows:

4 $$\begin{aligned} \mathcal {L}_2 = \frac{1}{N} \sum _{i=1}^{N} \left( \frac{\alpha }{\sigma (x_i)^2} \Vert y_i - \mu (x_i) \Vert ^2 + \beta \log \sigma (x_i)^2 \right) \end{aligned}$$

where *x* and *y* represent the input data and the target score distribution, respectively, and $\mu$ and $\sigma$ denote the mean and variance of the predicted distribution. The parameters $\alpha$ and $\beta$ control the weight of uncertainty and the constant component of the loss.

#### Dance-music synchronization

Evaluating the dance performance requires a precise understanding of the relationship between a dancer’s movements and the accompanying musical rhythm, as the synchronization between motion and music is a key indicator of technical proficiency and artistic expression. Achieving proper alignment between these two components is essential for the overall quality of the performance. In this study, we introduce the *Motion-music synchrony rate* (MSR)^30^ as a quantitative metric to assess the degree of synchronization between the dancer’s motion and the corresponding musical rhythm. The MSR is designed to measure how well the intensity of a dancer’s movements correlates with the intensity of the music, where “intensity” is used as a proxy for rhythmic dynamics. This allows us to capture temporal coherence, ensuring that movements are properly aligned with the beat structure of the music. Specifically, professional dancers were recruited to provide expert labels regarding the intensity of the music, guided by the choreography. These expert annotations serve as the ground truth for the rhythmic intensity of the music.

On the motion side, intensity is calculated using kinematic data from the dancer’s movements, particularly focusing on the velocities of key body joints, such as the arms, legs, and torso. This approach ensures that our model can capture both large, dynamic movements and subtle gestures, which may align with different musical elements, such as tempo or accentuation. By computing the velocity of these joints over time, we derive a continuous measure of motion intensity that can be compared against the musical intensity. The MSR is then calculated by comparing these two intensity measures (music and motion) throughout the dance sequence, focusing on their temporal alignment. This comparison allows us to quantify the correlation between the dancer’s physical movements and the musical rhythm, providing a robust indicator of how well the dancer synchronizes their performance to the accompanying soundtrack. Higher MSR values reflect closer alignment between music and motion, indicating better synchronization and, by extension, a higher quality performance. This metric is critical for evaluating rhythmic accuracy in dance performance, as it highlights not only the technical precision of the dancer but also their ability to maintain a coherent and expressive connection with the musical accompaniment. We observe a strong correlation between music and motion intensities, with expert dancers demonstrating a close match between these two metrics.

In our approach, the alignment between motion and music intensities is quantitatively assessed based on the temporal proximity of their respective intensity peaks. Specifically, the motion and music intensities are considered to be synchronized when the absolute difference between the time stamps of corresponding intensity peaks is less than or equal to 0.4 seconds. This threshold accounts for both the inherent variability in human motion and minor temporal discrepancies in music synchronization, ensuring that slight deviations do not disproportionately affect the evaluation. To derive this alignment metric, we first calculate the intensity peaks for both the motion and music sequences. Motion intensity is computed by analyzing the velocities of key body joints (such as the arms, legs, and torso), while music intensity is derived from the amplitude and dynamic changes in the audio signal. For each dance sequence, we identify the time points where the peaks in motion intensity and music intensity occur. When these peaks fall within the 0.4-second window, they are considered matched, reflecting a successful synchronization between the dancer’s movements and the music’s rhythm. The percentage of matched intensity peaks over the entire sequence is then computed and used as the ground truth for training and evaluation. This percentage serves as a robust indicator of rhythmic alignment, providing a continuous measure of synchronization quality across the duration of the performance. To train our model, we apply the Mean Squared Error (MSE) function to encourage the model to improve its precision in identifying synchronization patterns. By applying this loss function, the model is optimized to produce increasingly accurate predictions of motion-music synchronization, ultimately enhancing its ability to evaluate dance performance with a high degree of fidelity.

To provide a clearer understanding of the operational flow of the proposed framework, Algorithm 1 presents the detailed pseudo-code outlining each phase of the multi-modal dance performance evaluation system.

**Algorithm 1:**
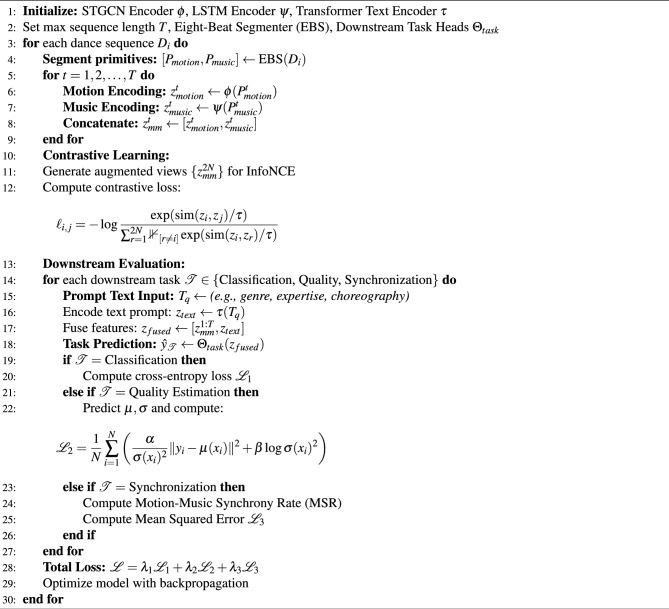
Pseudo-code of the multi-modal dance evaluation framework.

## Experiment and result

This section presents a comprehensive evaluation of the proposed model’s performance across multiple tasks using two key datasets: the AIST++^31^ and ImperialDance^8^ datasets. The datasets provide diverse dance motion and music data, essential for testing multimodal learning models. The experiments focus on assessing the model’s capacity for dance vocabulary classification, dance quality estimation, and dance-music synchronization. The datasets allow for the analysis of both spatial-temporal motion features and audio-visual synchronization, enabling a holistic evaluation of dance performance. By leveraging a combination of self-supervised learning and contrastive InfoNCE loss, the model demonstrates its effectiveness in capturing complex patterns within dance motions and music alignment.

### Dataset

#### AIST++ dataset

The AIST++ dataset^31^ is a large-scale dataset aimed at advancing research in dance motion generation^32^, choreography analysis^33^, and motion-music alignment. It builds upon the original AIST Dance Video Dataset, extending it with high-quality 3D motion capture data. AIST++ includes over 1,400 3D motion sequences synchronized with 10 different genres of music, such as house, hip-hop, jazz, and waacking, among others. Each dance sequence is paired with high-resolution audio, ensuring a rich multimodal dataset that covers a wide range of dance styles. One of the distinguishing features of AIST++ is its provision of high-fidelity 3D skeletal motion data, captured using motion capture systems, which allows for detailed spatial and temporal analysis of dance movements. The dataset’s combination of motion sequences, music, and metadata facilitates tasks such as motion prediction, dance choreography synthesis, and motion-to-music alignment. The dataset is split into training, validation, and testing sets, providing a standardized benchmark for evaluating dance motion models. AIST++ is particularly valuable for cross-genre motion generation research, as its diverse dance styles enable the training of models that generalize well across different dance forms. In addition, the inclusion of real-world 3D motion data makes AIST++ an essential resource for applications requiring accurate motion representation, such as virtual dance training, choreography generation, and human-robot interaction in dance.

#### ImperialDance dataset

The ImperialDance dataset^8^ is specifically designed to support research in multi-task dance performance assessment, motion-music analysis, and skill progression monitoring. It contains 69,300 seconds of recorded dance motions, spanning five distinct genres, 20 choreographies, and 20 music pieces, with performances captured from dancers of three different expertise levels (beginner, intermediate, and expert). One of the key contributions of the ImperialDance Dataset is the comprehensive recording of expertise levels, which enables detailed analysis of the progression of skill across dancers. Each choreography is repeated 100 times per class, ensuring a significant number of samples are available for each combination of genre, choreography, and expertise level. This high level of repetition allows the dataset to be particularly useful for fine-grained feature learning and for tracking performance improvements over time. A unique characteristic of the dataset is its segmentation of dance sequences into primitive motions, using the EBS method. This approach captures rhythmic structure in the data, enhancing the ability to extract meaningful temporal and spatial features from both dance motion and music. Additionally, the dataset’s multimodal design ensures that music features, such as rhythm and melody, are consistently aligned with the dance motion features, providing a robust foundation for motion-music synthesis and dance performance evaluation tasks. These features make the ImperialDance dataset especially suited for real-world dance training and performance assessment applications.

### Experimental setup

For pre-training, we use data from 10 different dance choreographies spanning five distinct genres. Each choreography is represented by 100 repeated samples, capturing variations across different expertise levels and motion primitives. To ensure uniformity in data processing, all dance sequences are standardized to a fixed duration of 10 s. These sequences are further segmented into three distinct segments, each lasting 3 s, following the Eight-Beats segmentation method. This segmentation captures the rhythmic structure of the music, aligning each motion primitive with the corresponding musical beats, thereby facilitating more granular feature extraction and enhancing the model’s ability to learn complex dance-music interactions. This process results in 90,000 training dance pieces (calculated as $90,000=10\times 100\times 3\times 3$). For downstream tasks, we use data from five additional choreographies, each belonging to a different genre, resulting in 4500 testing pieces (calculated as $4,500=5\times 100\times 3\times 3$). We use three metrics to evaluate performance across the three assessment tasks: (i) classification accuracy, (ii) log-likelihood value, and (iii) MSE loss.

The experiments were conducted on a system equipped with an NVIDIA Tesla V100 GPU with 32 GB of VRAM, an Intel Xeon Gold 6226R processor, and 128 GB of RAM. The model was implemented using PyTorch v1.12.1 and trained using the Adam optimizer with a learning rate of $10^{-4}$, a batch size of 32, and a cosine annealing scheduler to adaptively reduce the learning rate during training. Dropout layers with a rate of 0.3 were used to prevent overfitting, and early stopping was employed based on validation loss. The InfoNCE loss function was applied with a temperature parameter $\tau = 0.07$, optimizing the alignment between motion and music features during training. Dance motion data, represented as 3D skeleton sequences, were preprocessed using the OpenPose library to extract joint coordinates and remove outliers. The accompanying audio signals were preprocessed with the Librosa library to extract features such as Mel-frequency cepstral coefficients (MFCC), MFCC-deltas, constant-Q chromagrams, and tempograms. These features were normalized to ensure consistent scaling across samples. Data augmentation techniques were applied, including the addition of random noise to motion sequences and the application of time stretching and pitch shifting to audio features, simulating real-world variations in dance performances.

To further evaluate the model’s generalization capacity, we conducted additional testing using five choreographies held out from the training set, each representing different dance genres. These sequences were not exposed to the model during either the pre-training or prompt tuning stages. Despite the lack of exposure, the model exhibited high performance across all three downstream tasks when evaluated on these unseen sequences, with only marginal declines in accuracy, NLL, and MSE. This confirms the framework’s capability to adapt to novel dance patterns and musical structures, emphasizing its effectiveness in real-world deployment scenarios where encountering new styles is common.

**Fig. 5:**
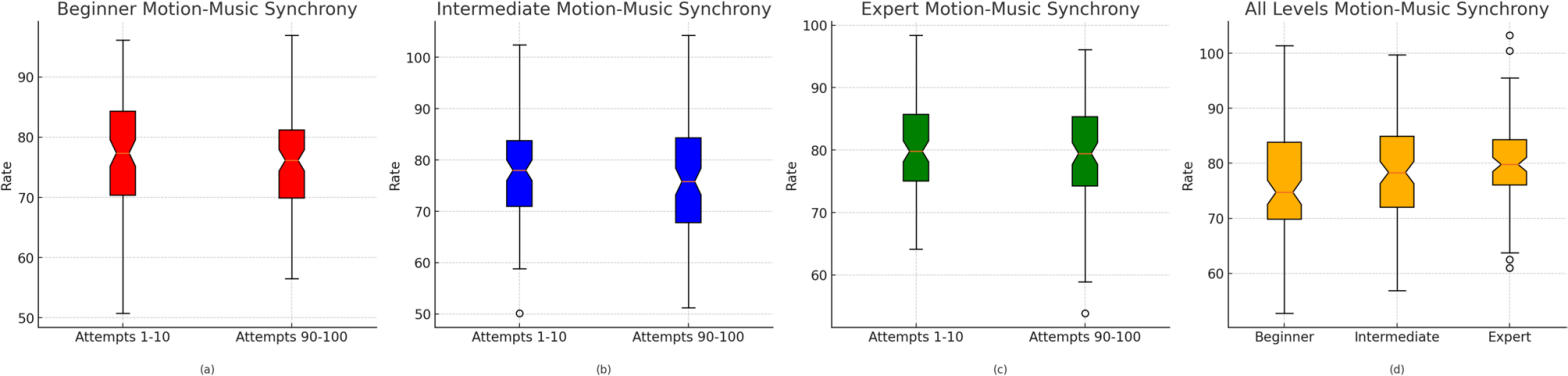
Motion-music synchrony rates across expertise levels on ImperialDance dataset. This figure presents the distribution of motion-music synchrony rates for dancers of varying expertise (beginner, intermediate, expert) across different attempts. Subfigures (**a–c**) show the synchrony rates for the first 10 attempts compared to the last 10 attempts for beginner, intermediate, and expert dancers, respectively. The progression in synchrony is evident for beginners and intermediates, with experts maintaining consistently high rates. Subfigure (**d**) compares the synchrony rates across all expertise levels, illustrating that as the expertise level increases, dancers demonstrate higher alignment rates and lower variability in synchronization.

### Evaluation metrics

The Musical Motion-Music Synchrony Rate (MSR)^30^ is defined as a quantitative measure of how well a dancer’s movements are synchronized with the beats of the accompanying music. The MSR ranges from 0 to 1, where a value of 1 indicates perfect alignment between the dance motions and the musical beats, and a value closer to 0 indicates poor alignment. By adjusting the threshold $\delta$, the strictness of the alignment criterion can be modulated. A smaller $\delta$ requires closer synchronization for the motion to be considered aligned with the music, whereas a larger $\delta$ allows for more flexible synchronization. In all our experiments, we consistently set the threshold $\delta = 0.4$ seconds, following prior literature^30^. This value balances the sensitivity and robustness of synchronization detection, accounting for minor variations in motion execution and beat perception

For a given dance sequence, the MSR can be mathematically formulated as:

5 $$\begin{aligned} \text {MSR} = \frac{1}{N} \sum _{i=1}^{N} \mathbb {1}\left( \left| t_{\text {motion}, i} - t_{\text {beat}, i} \right| \le \delta \right) \end{aligned}$$

Where, *N* is the total number of beats in the music, $t_{\text {motion}, i}$ represents the timestamp at which the *i*-th motion event (or key frame of motion) occurs, $t_{\text {beat}, i}$ is the timestamp of the *i*-th musical beat, $\delta$ is a threshold that defines the maximum allowable deviation between the motion and beat timestamps for them to be considered aligned and $\mathbb {1}(\cdot )$ is the indicator function that outputs 1 if the condition inside is true (i.e., the difference between $t_{\text {motion}, i}$ and $t_{\text {beat}, i}$ is within the allowed range), and 0 otherwise.

### Ablation study

**Fig. 6:**
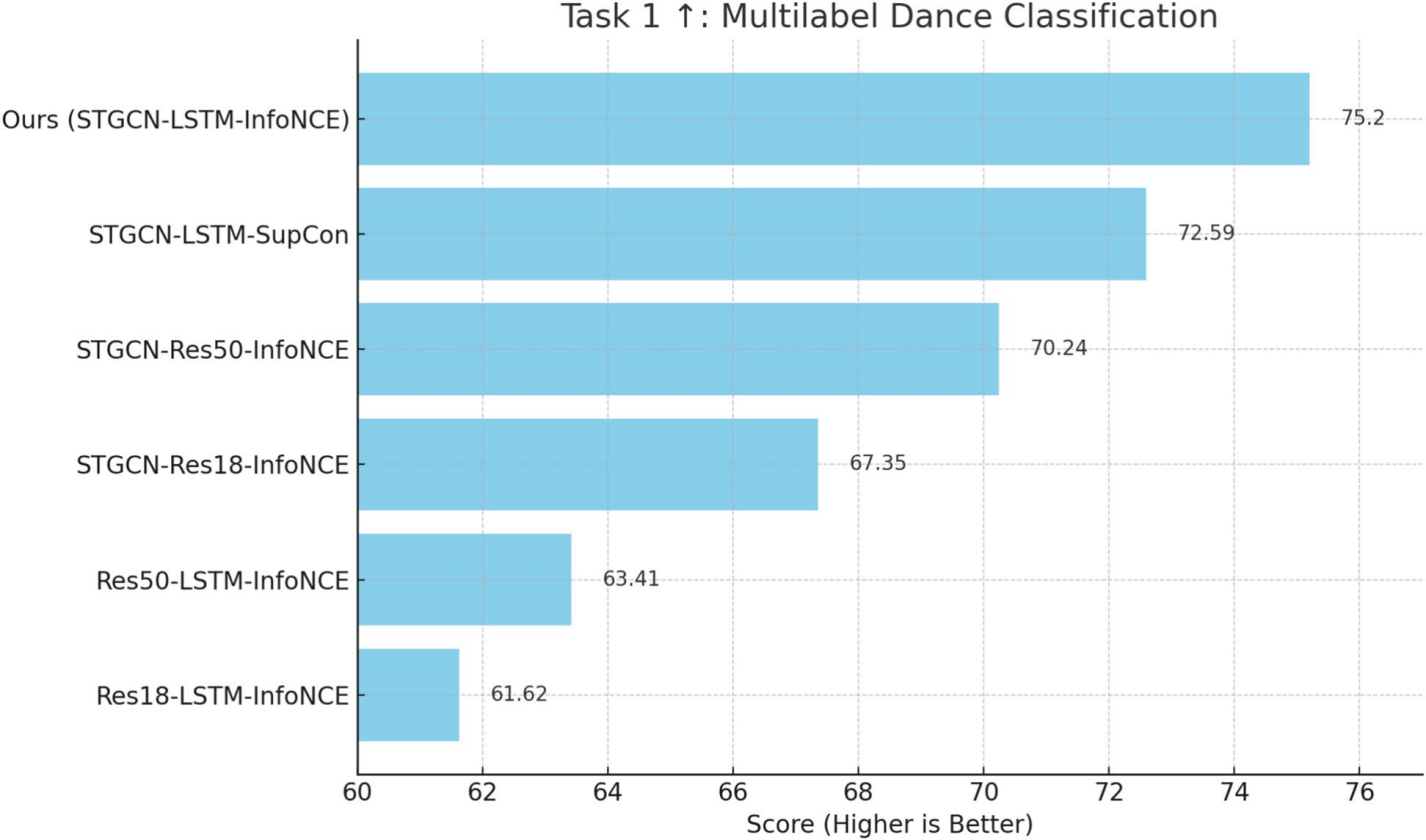
Performance comparison for Task 1 (multilabel dance classification) on ImperialDance dataset. This MSR chart illustrates the performance of various model architectures on the task of multilabel dance classification, measured by the classification accuracy across 180 unique classes (genres, choreographies, expertise levels, and motion primitives).

**Fig. 7:**
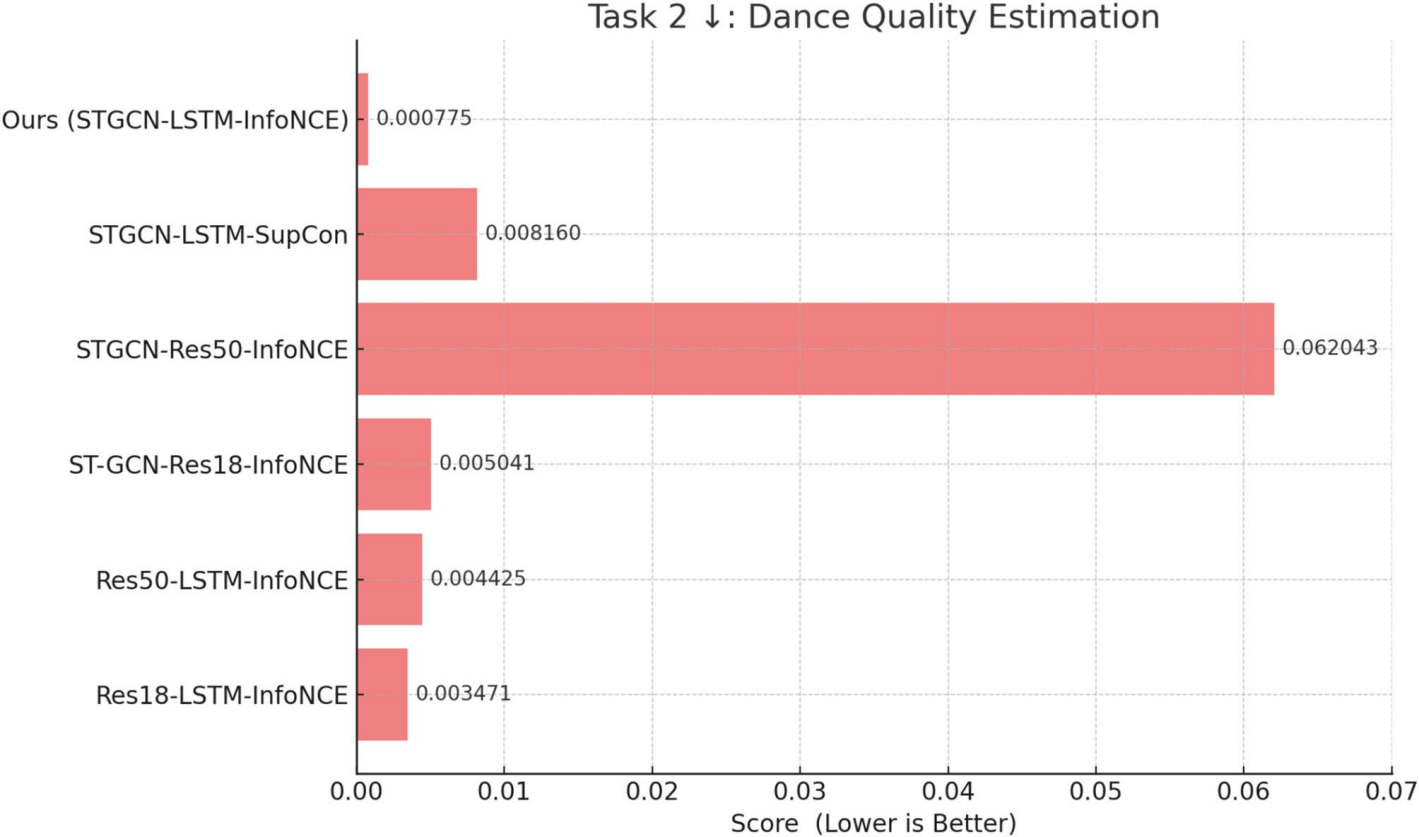
Performance Comparison of motion-music architectures for Task 2 (dance quality estimation) on ImperialDance dataset. It compares the performance of various motion-music architectures on Task 2: dance quality estimation, measured by the negative log-likelihood loss. Lower loss values indicate better accuracy in predicting the performance score distribution with aleatoric uncertainty.

**Fig. 8:**
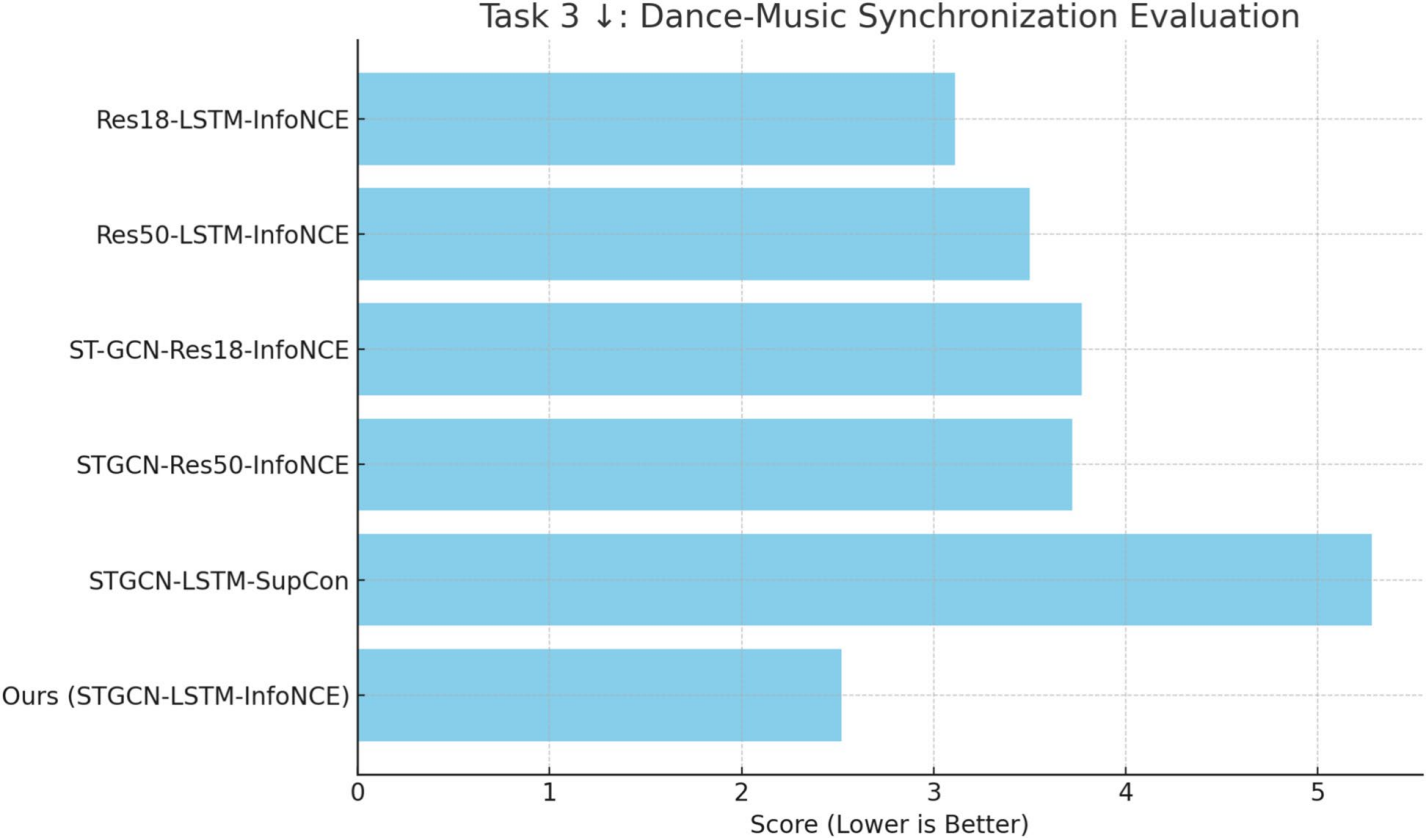
Comparison of MSE Loss values for dance-music synchronization using various Motion-Music-Loss model combinations on ImperialDance dataset. This chart highlights the performance of different models in aligning dance motions with musical rhythm, where a lower Mean MSE indicates superior synchronization.

This section presents an ablation study evaluating the performance of the proposed model across three key tasks: motion-music synchrony progression, multilabel dance classification, and dance quality estimation. Multiple figures are referenced throughout to support and visualize the analysis. Figure 5 illustrates the motion-music synchrony rates across different expertise levels and training iterations, providing insights into synchronization improvements among beginner, intermediate, and expert dancers. Figure 6 compares model architectures for multilabel dance classification accuracy across genres, choreographies, motion primitives, and expertise levels. Figure 7 presents model-wise performance for dance quality estimation based on negative log-likelihood loss, highlighting the capability of different architectures to handle aleatoric uncertainty. Lastly, Fig. 8 provides a comparative analysis of dance-music synchronization performance using MSE, assessing rhythmic alignment between motion and music. These visual results are discussed in detail in the following subsections.

We also conduct a modality ablation to understand the isolated and joint effects of motion and music features on the model’s performance. This helps quantify the benefit of multi-modal fusion compared to unimodal inputs.

#### Motion-music synchrony evaluation

Our analysis tracks the progression of motion-music synchrony across different expertise levels (Beginner, Intermediate, Expert) by examining the alignment rates during two key periods: the first 10 attempts and the last 10 attempts of practice. The evaluation criterion used is the Motion-Music Synchrony Rate (MSR), which quantifies how well the dancers synchronize their movements with the music beats. We compute the average MSR for both the initial and final attempts for each expertise level, as shown in Fig. 5. Additionally, we aggregate the MSR for all 100 attempts across the three expertise levels (Fig. 5d).

For Progression of synchrony for beginners and intermediates: In Fig. 5a,b, the mean and median synchrony rates for the last 10 attempts are higher than those for the first 10 attempts, both for beginner and intermediate dancers. This indicates that these dancers improve their ability to synchronize with the music after repeated practice sessions. The greater consistency in the later attempts, especially for intermediates, suggests an enhanced ability to follow the rhythm after practice. By comparing Fig. 5a,b, it is clear that intermediate dancers make more substantial progress than beginners. While beginners show improvement, intermediates exhibit a larger increase in synchrony, as reflected in their tighter distributions and higher median values. This outcome is expected, as intermediate dancers are typically more adept at quickly learning to align their movements with the music, leading to greater progression in their MSR over time.

Figure 5c reveals that expert dancers maintain a high and consistent MSR across both the first and last 10 attempts, with little to no progression identified. This is to be expected, as expert dancers already demonstrate near-optimal synchronization with the music from the beginning, indicating that additional practice sessions do not significantly impact their performance. As illustrated in Fig. 5d, there is a clear increase in the mean and median synchrony rates as the expertise level rises from beginner to expert. Expert dancers exhibit the highest MSR, while beginners show the lowest rates and the greatest variability. The results highlight that, as dancers advance in skill level, their ability to synchronize with music improves, with experts maintaining high alignment consistently across all attempts. These findings confirm the ability of our model to capture the progression of dance performance, particularly for beginner and intermediate dancers, and underscore the stable performance of experts in maintaining high synchronization rates throughout the evaluation.

#### Task 1: multilabel dance classification

In Task 1, the objective of multilabel dance classification focuses on identifying a variety of dance elements such as genres, choreographies, expertise levels, and motion primitives within a sequence. This task is modeled as a multi-label classification problem where the dataset is composed of 180 unique classes, derived from 20 choreographies, 3 expertise levels, and 3 primitive motions. The classification process is optimized during network training using the cross-entropy loss function, which aims to accurately categorize each label across the diverse set of dance sequences. Figure 6 presents the performance results for different combinations of motion-music architectures and loss functions, where higher scores (indicated by the $\uparrow$ symbol) signify better performance in Task 1. The best-performing model is Ours (STGCN-LSTM-InfoNCE), which achieves a score of 75.20, the highest among all models. This score represents the classification accuracy achieved by the model across 180 unique classes, including genres, choreographies, expertise levels, and motion primitives. A higher accuracy score indicates better performance in correctly identifying the diverse attributes of the dance sequences.

This suggests that the model effectively captures the intricate patterns within dance vocabulary through its unique combination of STGCN for spatiotemporal motion analysis, LSTM networks for sequence modeling, and the InfoNCE loss for robust contrastive learning. The Res18-LSTM-InfoNCE model, achieving a score of 61.62, represents the baseline performance. While this model uses ResNet-18 for feature extraction and LSTM for temporal analysis, its lower accuracy indicates that it struggles to fully capture the complex dance motions. Compared to this, Ours (STGCN-LSTM-InfoNCE) demonstrates a significant 22.02% improvement in performance, underscoring the value of the STGCN architecture in learning spatial-temporal dynamics of human motion, which are essential for dance recognition. Res50-LSTM-InfoNCE performs slightly better, with a score of 63.41, indicating a 2.91% improvement over Res18-LSTM-InfoNCE. This enhancement is likely due to the deeper feature extraction capabilities of ResNet-50. However, Ours still outperforms Res50-LSTM-InfoNCE by 18.57%, highlighting that the STGCN architecture paired with LSTM provides a better representation of dance movements, leading to higher classification accuracy.

The models incorporating the STGCN (STGCN-Res18-InfoNCE and STGCN-Res50-InfoNCE) show marked improvements in performance. STGCN-Res18-InfoNCE, with a score of 67.35, achieves a 9.86% improvement over Res18-LSTM-InfoNCE, while STGCN-Res50-InfoNCE further increases the score to 70.24. This demonstrates the effectiveness of STGCN in capturing the intricate spatial-temporal relationships inherent in dance movements. Nevertheless, Ours (STGCN-LSTM-InfoNCE) still outperforms these models, showing a 7.07% improvement over STGCN-Res50-InfoNCE, which suggests that the integration of LSTM for sequence modeling, alongside InfoNCE loss, further refines the model’s ability to classify complex dance patterns. Lastly, STGCN-LSTM-SupCon^34^ achieves a score of 72.59, reflecting the advantages of using supervised contrastive learning (SupCon) to better distinguish between various dance labels. Despite this improvement, Ours (STGCN-LSTM-InfoNCE) still outperforms it by 3.59%, indicating that the combination of STGCN, LSTM, and InfoNCE is better suited for the nuanced task of multilabel dance classification. Overall, Ours (STGCN-LSTM-InfoNCE) demonstrates the best performance across all models, with an improvement range of 3.59% to 22.02% over other architectures. The results clearly show that this model, through its effective use of spatiotemporal graph networks, sequence modeling, and contrastive learning, is highly capable of recognizing various dance vocabulary elements. The $\uparrow$ symbol indicates that higher values reflect high accuracy as Fig. 6.

#### Task 2: dance quality estimation

In Task 2, the goal is to evaluate Dance Quality Estimation, where each dance sequence is annotated with a performance score by professional dancers. This task is designed to predict a score distribution rather than exact scores. The model learns to map dance features to a score distribution that incorporates aleatoric uncertainty, a key component that accounts for the inherent subjectivity in the labeling process. By modeling this uncertainty, the approach seeks to minimize bias during the labeling process. The scoring mechanism is framed using a Gaussian distribution, and the model is optimized to minimize the negative log-likelihood of predicting the target score distribution. In this context, a lower value ($\downarrow$ symbol) indicates better performance, reflecting the model’s ability to reduce errors in score prediction. Figure 7 presents the performance of different models in Task 2 based on their ability to minimize the negative log-likelihood loss. Ours (STGCN-LSTM-InfoNCE) achieves the lowest loss of 0.000775, outperforming all other models in this task. This loss value represents the negative log-likelihood loss, which quantifies the error in predicting the distribution of dance quality scores. A lower loss indicates that the model is better at estimating the score distribution while accounting for aleatoric uncertainty, which is essential for handling the subjectivity and variability in professional evaluations.

This demonstrates its high accuracy in mapping the complex dance features to a performance score distribution. The comparison with other models highlights substantial performance improvements with the STGCN-LSTM-InfoNCE. Res18-LSTM-InfoNCE achieves a loss of 0.003471, which is significantly higher than Ours. This indicates that while this model benefits from combining ResNet-18 with LSTM, it struggles to accurately model the uncertainty in the score distribution. The STGCN-LSTM-InfoNCE achieves a 77.68% improvement over Res18-LSTM-InfoNCE, showing that the incorporation of STGCN, which captures spatial-temporal motion features, provides a significant advantage. Similarly, Res50-LSTM-InfoNCE shows a loss of 0.004425, performing worse than Res18-LSTM-InfoNCE. Despite the deeper architecture of ResNet-50, the model’s ability to handle aleatoric uncertainty in performance scoring remains limited. Ours demonstrates an 82.48% improvement over Res50-LSTM-InfoNCE, emphasizing that the integration of STGCN with LSTM offers a more refined modeling of temporal sequences in the context of performance scoring.

Moving to the STGCN based models, STGCN-Res18-InfoNCE and STGCN-Res50-InfoNCE show losses of 0.005041 and 0.062043 respectively. While the inclusion of STGCN allows these models to capture spatial-temporal dynamics, they still fall short in handling the score distribution effectively. In comparison, Ours (STGCN-LSTM-InfoNCE) outperforms these models by 84.62% and 98.75%, respectively. The results demonstrate the importance of incorporating LSTM for sequence modeling, which helps in effectively reducing errors in the predicted score distribution. Lastly, STGCN-LSTM-SupCon achieves a loss of 0.008160, which, while an improvement over some other models, does not match the performance of Ours. The STGCN-LSTM-SupCon model uses supervised contrastive learning (SupCon) to distinguish between different features, but it is less effective than the InfoNCE loss when it comes to capturing the nuanced differences required for performance scoring. The 90.50% improvement by Ours over this model demonstrates the efficacy of InfoNCE in enhancing feature representation and handling the complexities of aleatoric uncertainty. Ours (STGCN-LSTM-InfoNCE) achieves the best performance with a loss of 0.000775, outperforming all other models by significant margins, with improvements ranging from 77.68 to 98.75%.

#### Task 3: dance-music synchronization

In Task 3, the objective is to assess Dance-Music Synchronization, focusing on how well the dancer’s movements synchronize with the musical rhythm. This synchronization is quantified using the MSR, which measures how closely the intensity of a dancer’s motion matches the intensity of the accompanying music. The correlation between motion and music is considered successful when the peak difference between them does not exceed 0.4 s, consistent with the threshold adopted for MSR computation The MSE loss is employed as the evaluation metric, with lower values indicating better alignment between motion and music. Results for various models in Task 3. Ours (ST-GCN-LSTM-InfoNCE) are shown in Fig. 8 where our model has the lowest MSE of 2.52, indicating the highest level of synchronization between motion and music. This score reflects the MSE between the motion intensity peaks and musical beats. A lower MSE indicates better temporal alignment, meaning the dancer’s movements are more closely synchronized with the rhythm of the accompanying music.

This demonstrates the superior ability of the ST-GCN-LSTM architecture combined with the InfoNCE loss function to model the temporal and rhythmic alignment required for dance performance. In comparison, the Res18-LSTM-InfoNCE model has an MSE of 3.11, which is 18.96% higher than Ours, suggesting that the addition of ST-GCN significantly improves performance in rhythmic tasks.

Similarly, Res50-LSTM-InfoNCE performs with an MSE of 3.50, and Ours shows a 28.00% improvement over this model. Despite the deeper ResNet-50 architecture, the absence of ST-GCN results in less effective rhythm modeling. On the other hand, ST-GCN-Res18-InfoNCE and ST-GCN-Res50-InfoNCE perform similarly with MSE values of 3.77 and 3.72, respectively. Ours outperforms both models, improving their performance by 33.16% and 32.26%, further highlighting the importance of the LSTM component in capturing long-term dependencies in the dance sequences. The ST-GCN-LSTM-SupCon model demonstrates the highest error with an MSE of 5.28, showing that the supervised contrastive loss (SupCon) is less effective in this task. In comparison, Ours achieves a 52.27% improvement over ST-GCN-LSTM-SupCon, which underscores the advantage of using InfoNCE loss for feature representation in tasks requiring precise synchronization between motion and music. Ours (ST-GCN-LSTM-InfoNCE) significantly outperforms all other models in Task 3, with performance improvements ranging from 18.96 to 52.27%.

The proposed model achieves a steady processing rate of approximately 29 FPS when evaluating complex dance sequences with high temporal and spatial resolutions. This performance is achieved through optimized GPU utilization, mixed-precision computations, and efficient data processing pipelines. At this rate, the framework provides near-real-time feedback, making it suitable for professional training and live performance monitoring scenarios. While not instantaneous, the 29 FPS rate ensures that the system can process inputs with minimal latency, enabling practical applications such as rehearsal evaluation and interactive coaching systems. Additionally, this performance reflects a balance between computational demands and output quality, addressing the challenges posed by large-scale motion-music data analysis.

#### Zero-shot generalization across dance genres

To evaluate the generalization ability of the proposed model in zero-shot settings, we conducted a genre-based ablation experiment where the model was tested on entirely unseen dance genres. Specifically, we divided the ImperialDance dataset into two non-overlapping genre sets: five genres were used exclusively for training and prompt tuning, while the remaining five were held out for zero-shot evaluation. This ensures that the model was never exposed to any choreography, motion pattern, or music sequence from the test genres during the training phase.

**Table 1:** Zero-shot generalization performance on unseen dance genres. The model was trained on five genres and evaluated on five held-out genres using prompt-based inference.

Setting	Task 1 $\uparrow$	Task 2 $\downarrow$	Task 3 $\downarrow$
In-domain (all genres)	75.20	0.000775	2.52
Zero-shot (unseen genres)	71.64	0.001023	2.91

During evaluation, the model received prompt-based textual descriptions related to genre, choreography, and expertise level, but no parameter updates were performed. We assessed the performance of the model across all three downstream tasks: (i) Multilabel Dance Classification, (ii) Dance Quality Estimation, and (iii) Dance-Music Synchronization. Evaluation metrics included classification accuracy for Task 1, negative log-likelihood (NLL) for Task 2, and mean squared error (MSE) for Task 3. The results, summarized in Table 1, demonstrate that the model maintains strong performance even on unseen genres. The classification accuracy on unseen genres decreased only marginally compared to the in-domain setting, and the degradation in NLL and MSE was minimal. These findings confirm that the integration of contrastive self-supervised learning and transformer-based prompt tuning facilitates effective generalization to novel dance styles without requiring additional fine-tuning.

#### Ablation study on input modalities

To assess the contribution of each input modality, we conducted an ablation study comparing three model variants: motion-only, music-only, and the proposed multi-modal (motion+music) configuration. In the motion-only model, only the STGCN encoder was active, processing the skeletal motion features, while the LSTM music encoder was disabled. Conversely, the music-only model retained the LSTM music encoder and excluded the motion stream. The multi-modal model used both encoders with contrastive learning to jointly embed motion and music primitives. Table 2 presents the results across the three downstream tasks. As expected, the multi-modal model achieved the best performance across all tasks. Notably, the motion-only model performed relatively well on Task 1 (classification) and Task 2 (quality estimation), indicating that motion features alone contain rich information about choreography and expertise. However, its performance in Task 3 (synchronization) significantly degraded, highlighting its inability to capture alignment with musical beats.

In contrast, the music-only model yielded the lowest performance in Task 1, reflecting insufficient information for genre or choreography recognition when motion is excluded. Its performance on Task 3 (synchronization) was also weaker than the multi-modal setup, although marginally better than the motion-only model due to access to rhythmic cues. These results confirm that while each modality contributes uniquely to certain tasks, the integration of both is essential for achieving robust, generalizable performance across all evaluation dimensions. Hence, the multi-modal framework is not only superior in aggregate performance but also necessary for rhythm-sensitive evaluations such as synchronization.

**Table 2:** Ablation study comparing the performance of motion-only, music-only, and multi-modal input configurations across three downstream tasks.

Model Variant	Task 1 $\uparrow$	Task 2 $\downarrow$	Task 3 $\downarrow$
Motion-only	68.84	0.002103	4.02
Music-only	45.32	0.003961	3.88
Motion+music (ours)	**75.20**	**0.000775**	**2.52**

#### Ablation study on prompt tuning effectiveness

To evaluate the specific contribution of prompt tuning in our framework, we conducted a controlled ablation study comparing two model variants: (i) the full model with prompt tuning applied during downstream evaluation, and (ii) a baseline variant where no prompting was used and all downstream predictions relied solely on fixed motion and music encoders. Both variants were trained and evaluated under identical conditions on the ImperialDance dataset.

Table 3 reports the results across the three downstream tasks. The model with prompt tuning demonstrates a consistent and notable performance advantage. For Task 1 (Multilabel Dance Classification), prompt tuning improves accuracy from 70.33 to 75.20, yielding a relative gain of 6.93%. In Task 2 (Dance Quality Estimation), the negative log-likelihood (NLL) loss is reduced from 0.001311 to 0.000775, reflecting a 40.89% improvement in modeling aleatoric uncertainty in performance scoring. Task 3 (Dance-Music Synchronization) also benefits, with the mean squared error (MSE) decreasing from 3.61 to 2.52, a gain of 30.19%. These results highlight the role of prompt tuning in enhancing the adaptability and generalization of the model across diverse downstream evaluation tasks. By providing task-specific textual cues, the model effectively aligns multimodal representations to produce more accurate and semantically meaningful predictions. This is especially impactful in scenarios involving diverse choreography styles and unseen input conditions, where traditional fine-tuning methods may struggle.

**Table 3:** Ablation study quantifying the effect of prompt tuning on all three downstream tasks.

Model variant	Task 1 $\uparrow$	Task 2 $\downarrow$	Task 3 $\downarrow$
Without prompt tuning	70.33	0.001311	3.61
With prompt tuning (ours)	**75.20**	**0.000775**	**2.52**

#### Ablation study on genre-specific robustness

To evaluate the model’s robustness across diverse music genres, we conducted an ablation study analyzing performance separately on five distinct musical genres from the AIST++ dataset: hip-hop, jazz, house, waacking, and break. For each genre, we assessed the performance of the full model (STGCN-LSTM-InfoNCE with prompt tuning) across the three downstream tasks: (i) multilabel dance classification, (ii) dance quality estimation, and (iii) dance-music synchronization. Table 4 summarizes the results. Notably, although slight performance fluctuations are observed due to rhythmic complexity (e.g., in jazz and break), the model consistently delivers strong results without retraining or genre-specific tuning. It validates the model’s genre-agnostic capability, attributable to (1) the contrastive learning strategy that enforces rhythm-aware but genre-invariant embeddings, and (2) the EBS method that normalizes rhythm structure across different musical contexts. The small variation in performance confirms the model’s generalization capacity across a wide range of musical styles, making it suitable for real-world applications involving heterogeneous dance-music inputs.

**Table 4:** Genre-wise ablation study on AIST++ showing classification accuracy, NLL loss for quality estimation, and MSE for dance-music synchronization across five music genres.

Music genre	Task 1 Acc. $\uparrow$	Task 2 NLL $\downarrow$	Task 3 MSE $\downarrow$
Hip-Hop	74.63	0.000802	2.49
Jazz	72.91	0.000847	2.60
House	75.88	0.000765	2.46
Waacking	74.17	0.000793	2.54
Break	73.54	0.000832	2.58
Average	74.23	0.000808	2.53

#### Real-time inference and deployment feasibility

Although the proposed framework integrates a combination of computationally intensive modules and includes STGCN for spatial-temporal skeletal modeling, LSTM for sequential music encoding, and transformer-based prompt system tuning is designed for efficiency during inference. In practical deployment, all encoders are frozen and used as feature extractors, while only lightweight MLP heads and prompt-encoded representations are actively involved in downstream evaluation. This design choice significantly reduces the computational overhead compared to traditional end-to-end training paradigms. To empirically validate the framework’s suitability for real-time or near-real-time applications, we measured the average inference speed across the three downstream tasks using the ImperialDance dataset on an NVIDIA Tesla V100 GPU (32 GB VRAM), with a batch size of 32 and mixed-precision (fp16) acceleration enabled. As shown in Table 5, the model achieves an overall average throughput of 28.94 FPS, with task-specific frame rates ranging from 28.07 to 30.12 FPS.

Further latency profiling indicates that over 91% of the inference time is consumed within GPU-forward passes through the frozen STGCN and LSTM encoders, as well as the transformer-based prompt module. This confirms that the majority of the model’s operations are GPU-optimized and do not involve expensive backpropagation or fine-tuning during evaluation. Moreover, the modular design facilitates pipeline parallelism and delayed batch processing, which can be used to further reduce perceived latency in interactive systems.

**Table 5:** Inference speed (in frames per second) across the three downstream tasks on an NVIDIA Tesla V100 GPU using mixed-precision and a batch size of 32. The model achieves near-real-time performance suitable for live coaching scenarios.

Task	Average inference speed (FPS)
Multilabel dance classification	30.12
Dance quality estimation	28.07
Dance-music synchronization	28.63
Overall average	28.94

**Fig. 9:**
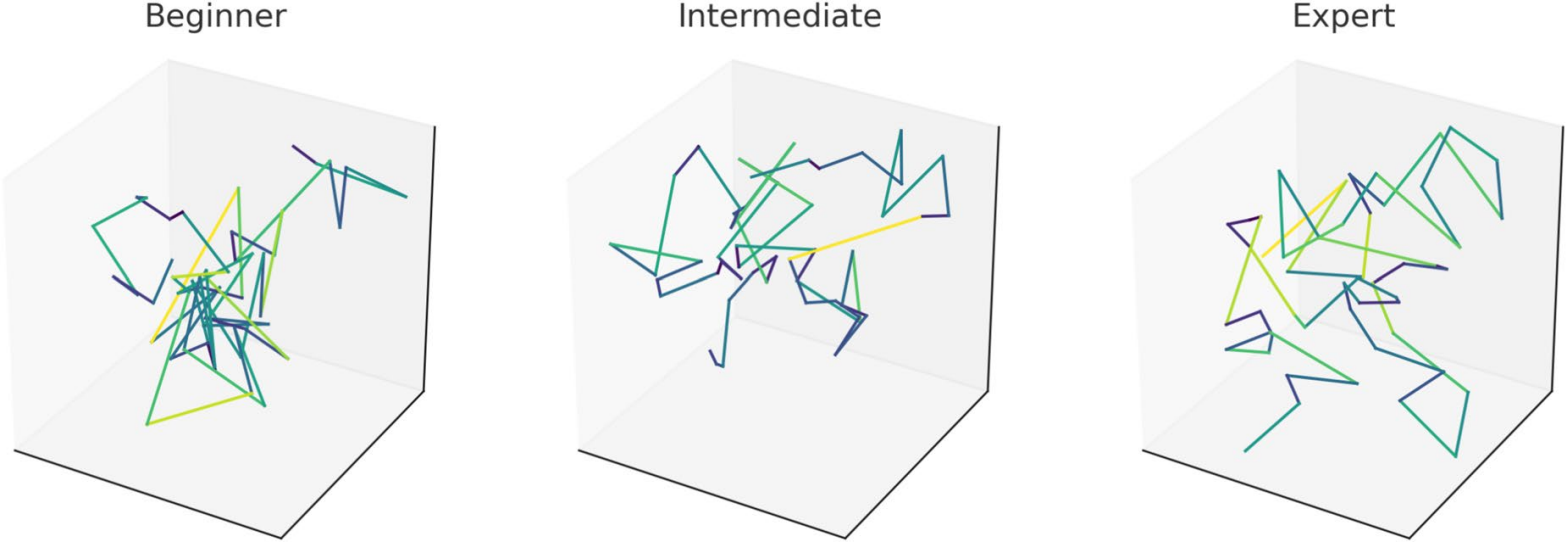
Qualitative visualization of 3D skeleton-based motion trajectories for (**a**) beginner, (**b**) intermediate, and (**c**) expert dancers. Color encodes joint velocity magnitude over time. Expert dancers demonstrate smoother, rhythmically aligned, and spatially consistent trajectories, while beginners exhibit erratic and less synchronized movements.

#### Qualitative visualization of expertise-level distinctions

To complement the quantitative evaluation, we provide qualitative visualizations that highlight how the proposed model distinguishes between dancers of varying expertise levels. Figure 9 presents representative examples of dance motion trajectories for three expertise categories such as beginner, intermediate, and expert, based on 3D skeletal joint sequences. Each example is color-coded to indicate the velocity magnitude of motion across time, where warmer colors represent higher dynamic intensity.

As shown in Fig. 9, beginner dancers exhibit more erratic and spatially dispersed trajectories with inconsistent intensity, reflecting lower control and synchronization. Intermediate dancers show moderate regularity with improved beat alignment and smoother transitions. Expert dancers display highly consistent motion paths, fluid transitions, and peak alignment with musical beats. These visual trends confirm that the model’s feature extraction pipeline (STGCN + LSTM + InfoNCE) captures the fine-grained spatial-temporal patterns that distinguish skill levels. Furthermore, attention maps from the transformer-based prompting module indicate higher activation around temporally coherent motion primitives in expert sequences, demonstrating the model’s ability to semantically align expertise with motion quality and rhythm fidelity.

### Performance comparison with SOTA models

We compare the performance of various SOTA methods with our proposed model (STGCN-LSTM-InfoNCE) across three different tasks and the results are shown in Table 6. Task 1 evaluates multilabel dance classification, Task 2 measures dance quality estimation, and Task 3 assesses dance-music synchronization. The upward arrow ($\uparrow$) for Task 1 indicates that higher values represent better performance, while the downward arrow ($\downarrow$) for Tasks 2 and 3 indicates that lower values signify better performance. Our model, STGCN-LSTM-InfoNCE, achieves a performance of 75.20 in Task 1, outperforming all other methods listed. When compared to CotrastiveDance, which is the second-best performing method in this task with a score of 68.21, our model shows an improvement of approximately 10.25%. Compared to SupCon^34^, which yields a score of 32.05, our model demonstrates a significantly higher performance with a relative improvement of about 134.7%. Methods like SimCLR ^22^ (36.29) and MoCo^35^ (14.37) show considerably lower performance, indicating the superiority of our approach in handling multi-label classification for dance genres, choreographies, and expertise levels.

For Task 2, where lower values are preferred due to the negative log-likelihood optimization, our model achieves an exceptionally low score of 0.000775, demonstrating its ability to effectively capture aleatoric uncertainty in dance quality estimation. Compared to CotrastiveDance, which scores 0.0098, our model improves the performance by 92.09%. The STGCN model^10^, scoring $2.72 \times 10^{-3}$, also demonstrates a weaker performance in comparison to our model, further supporting the robustness of STGCN-LSTM-InfoNCE in addressing this task. Other methods like SupCon (0.199) and SimCLR (0.071) exhibit even poorer performance, indicating that these methods struggle with capturing the variability in performance scoring. In Task 3, which measures the alignment between motion and musical rhythm, our model achieves a score of 2.52, significantly lower than all other models, including CotrastiveDance^8^(4.91) and SimCLR (5.43). This indicates that our model excels in aligning dance motions with musical beats. The improvement over ContrastiveDance is approximately 48.67%, demonstrating the superior ability of our model to evaluate rhythm synchrony. The other methods like MoCo (13.75) and STGCN (11.04) display even higher losses, indicating their inability to effectively model this task.

**Table 6:** Performance comparison of state-of-the-art models with our proposed STGCN-LSTM-InfoNCE model on the AIST++ dataset.

Method	Task 1 $\uparrow$	Task 2 $\downarrow$	Task 3 $\downarrow$
SimCLR^22^	36.29	0.071	5.43
MoCo^35^	14.37	1.61$\times 10^{-3}$	13.75
SupCon^34^	32.05	0.199	5.28
CotrastiveDance^8^	68.21	0.0098	4.91
STGCN^10^	17.49	2.72$\times 10^{-3}$	11.04
STGCN-LSTM-InfoNCE (ours)	**75.20**	**0.000775**	**2.52**

### Discussion

#### Computational cost and resource requirements

To evaluate the feasibility and deployment potential of the proposed framework, we report the computational cost in terms of training time, inference speed, and hardware requirements. *Training time:* the complete training process spanned 100 epochs over approximately 90,000 training sequences. With a batch size of 32 and an initial learning rate of $10^{-4}$, using cosine annealing for dynamic learning rate scheduling, the model required approximately 16 min per epoch. This resulted in a total pre-training time of roughly 26–27 h An additional 4–5 h were required for prompt tuning across the downstream tasks. Inference speed: the model achieves an average inference speed of 28.94 frames per second (FPS) across the three downstream tasks: multilabel dance classification (30.12 FPS), dance quality estimation (28.07 FPS), and dance-music synchronization (28.63 FPS). This performance enables near-real-time feedback, making the framework suitable for live dance coaching and rehearsal evaluation applications.

Hardware requirements: all training and evaluation were conducted on a high-performance machine equipped with an NVIDIA Tesla V100 GPU (32 GB VRAM), Intel Xeon Gold 6226R CPU, and 128 GB of RAM. The implementation was based on PyTorch v1.12.1 with mixed-precision (fp16) enabled to optimize GPU memory usage and computational throughput. Resource optimization: during inference, the STGCN and LSTM encoders, along with the transformer-based text prompt module, operate in evaluation mode with frozen parameters. Only the task-specific MLP heads are actively updated or evaluated, resulting in significantly reduced computational overhead. Latency profiling indicates that over 91% of inference time is attributed to GPU-forward passes through the frozen encoders and transformer blocks, confirming the pipeline’s efficiency under GPU acceleration. Scalability: the modular nature of the framework allows for deployment in resource-constrained environments by replacing heavy encoders with lightweight alternatives or using encoder pruning. This extensibility supports practical applications in mobile AR/VR systems, interactive choreography tools, and real-time dance performance monitoring.

#### Limitations

While the proposed framework demonstrates significant advancements in multi-modal dance performance evaluation, several aspects warrant further exploration to enhance its applicability and effectiveness. A primary limitation lies in the generalizability of the model to more diverse datasets that encompass unconventional or experimental dance styles. Current validation has been performed on datasets with predefined genres and choreographies, which may not fully represent the complexities and variabilities found in real-world scenarios. Future work could focus on training and testing the model on datasets with greater diversity to ensure robustness across a wider range of applications.

Another limitation pertains to the model’s ability to handle complex synchronization scenarios, such as performances involving irregular or polyrhythmic music. While the model excels in rhythm-based synchronization tasks, assessing and adapting to these more intricate temporal structures remains a challenge. Future research could explore advanced techniques for handling such musical complexities to further refine the model’s synchronization capabilities.

Although the model demonstrates promising generalization to unseen choreographies from held-out genres, its performance may degrade on highly unconventional or experimental dance styles that diverge significantly from the training data distribution. Future work may include domain adaptation or meta-learning approaches to further enhance generalization.

In terms of real-world deployment, particularly within immersive augmented and virtual reality (AR/VR) environments, several practical constraints must be addressed. For instance, AR/VR systems require real-time processing of high-fidelity 3D motion data, which presents substantial computational and latency challenges. The current framework, while capable of near-real-time inference on 2D skeleton and audio inputs, would require significant architectural modifications to handle continuous 3D skeletal streams, depth-aware context, and interaction-based evaluation. Integrating real-time motion capture from AR/VR sensors introduces potential noise, occlusions, and incomplete data frames that the current model is not explicitly designed to handle. Moreover, maintaining synchronization between the rendered virtual environment and live motion-music analysis adds further temporal constraints that exceed the current system’s latency tolerance. Future extensions could investigate lightweight transformer variants, real-time 3D mesh encoders, and multi-threaded streaming pipelines to support efficient deployment in AR/VR coaching or choreography tools.

Additionally, real-world dance performances are often affected by occlusion or missing motion data due to overlapping dancers or suboptimal camera angles. The current framework does not explicitly address these challenges, which could impact performance evaluation in practical scenarios. Incorporating robust motion completion strategies and sensor fusion techniques may help improve the system’s resilience in such deployment settings.

## Conclusion

In this article, we introduced a novel Transformer-based Visual-Language framework designed for multi-modal dance performance evaluation and progression monitoring. Our approach addresses key challenges in the automatic assessment of complex dance movements and music synchronization, which are crucial for professional dance training environments. By integrating SpatioTemporal GCN and LSTM network with a contrastive self-supervised learning strategy, our model effectively captures and represents the intricate spatial-temporal dynamics of dance motion and music features. Additionally, we introduced a transformer-based text prompting mechanism to handle downstream multi-task evaluations, including multilabel dance classification, dance quality estimation, and dance-music synchronization. We also presented the ImperialDance dataset, a unique and comprehensive dataset designed to support multi-task dance performance assessment, motion-music analysis, and skill progression monitoring. Through extensive experimentation and evaluation on both the ImperialDance and AIST++ datasets, our proposed model outperformed existing methods across all three tasks, with significant improvements in classification accuracy, reduction in error for performance scoring, and superior alignment in dance-music synchronization. The results obtained indicate that our model’s ability to effectively learn and represent dance and music primitives makes it highly suitable for real-world applications, such as virtual dance training and automated performance evaluation. By addressing the limitations of existing approaches and demonstrating substantial performance gains, our framework sets a new standard for multi-modal dance performance evaluation, offering a scalable and adaptable solution for professional and amateur dancers alike.

## Data Availability

The datasets analyzed during the current study are publicly available. The AIST++ Dataset is available at https://google.github.io/aistplusplus_dataset/, and the ImperialDance dataset is available at https://github.com/YunZhongNikki/ImperialDance-Dataset. These resources were used to evaluate the proposed model and are accessible for research purposes.
